# 1-Oleoyl Lysophosphatidic Acid: A New Mediator of Emotional Behavior in Rats

**DOI:** 10.1371/journal.pone.0085348

**Published:** 2014-01-07

**Authors:** Estela Castilla-Ortega, Leticia Escuredo, Ainhoa Bilbao, Carmen Pedraza, Laura Orio, Guillermo Estivill-Torrús, Luis J. Santín, Fernando Rodríguez de Fonseca, Francisco Javier Pavón

**Affiliations:** 1 Unidad de Gestión Clínica de Salud Mental, Hospital Regional Universitario de Málaga, Instituto de Investigación Biomédica de Málaga (IBIMA), Málaga, Spain; 2 Departamento de Psicobiología y Metodología de las Ciencias del Comportamiento, Universidad de Málaga, Instituto de Investigación Biomédica de Málaga (IBIMA), Málaga, Spain; 3 Departamento de Psicobiología, Universidad Complutense de Madrid, Madrid, Spain; 4 Unidad de Gestión Clínica de Neurociencias, Hospital Regional Universitario de Málaga, Instituto de Investigación Biomédica de Málaga (IBIMA), Málaga, Spain; Sapienza University of Rome, Italy

## Abstract

The role of lysophosphatidic acid (LPA) in the control of emotional behavior remains to be determined. We analyzed the effects of the central administration of 1-oleoyl-LPA (LPA 18∶1) in rats tested for food consumption and anxiety-like and depression-like behaviors. For this purpose, the elevated plus-maze, open field, Y maze, forced swimming and food intake tests were performed. In addition, c-Fos expression in the dorsal periaqueductal gray matter (DPAG) was also determined. The results revealed that the administration of LPA 18∶1 reduced the time in the open arms of the elevated plus-maze and induced hypolocomotion in the open field, suggesting an anxiogenic-like phenotype. Interestingly, these effects were present following LPA 18∶1 infusion under conditions of novelty but not under habituation conditions. In the forced swimming test, the administration of LPA 18∶1 dose-dependently increased depression-like behavior, as evaluated according to immobility time. LPA treatment induced no effects on feeding. However, the immunohistochemical analysis revealed that LPA 18∶1 increased c-Fos expression in the DPAG. The abundant expression of the LPA_1_ receptor, one of the main targets for LPA 18∶1, was detected in this brain area, which participates in the control of emotional behavior, using immunocytochemistry. These findings indicate that LPA is a relevant transmitter potentially involved in normal and pathological emotional responses, including anxiety and depression.

## Introduction

Lysophosphatidic acid (LPA; 1-acyl, 2-hydroxy-sn-glycero-3-phosphate) species are small signaling glycerophospholipids widely distributed throughout body tissues, including cerebrospinal fluid and plasma [1]–[3]. LPA species are produced through two primary metabolic pathways in serum and cell membranes [4], and the most abundant LPA forms are 1-oleoyl-LPA (LPA 18∶1), 1-palmitoyl-LPA (LPA 16∶0) and 1-linoleyl-LPA (LPA 18∶2) [5]. In the last decade, it was revealed that LPA acts through a complex family of G protein-coupled receptors (LPA_1−6_), widely distributed throughout the cardiovascular system, immune system, gut, lung, endocrine organs and brain [2], [6]–[9]. As a signaling molecule, LPA plays a role in multiple biological processes, ranging from cell biology (i.e., cell differentiation, survival and migration) to systemic roles (i.e., regulation of blood pressure, immunocompetence or reproduction) [2], [8]–[9], although the effects of each LPA species might depend on its affinity for the different LPA receptors [10].

Despite the numerous biological roles attributed to LPA, our knowledge of the role of this compound in the central nervous system (CNS) is just starting to emerge. LPA receptors are present in the developing brain, showing a dense presence of LPA_1_ and LPA_2_ receptors in the proliferative cortical ventricular zone [2], [6], [11]–[12]. At this stage, LPA serves as a signal for progenitor cell differentiation, survival and migration, and this compound controls developing neuron excitability through the direct regulation of ionic conductances [11], [13]–[14]. LPA might also regulate synapse formation during development [15], consistent with the fact that small Rho kinases, the primary cytoskeleton regulators, are effectors of LPA receptors [7], [16]. Moreover, when the ventricular zone disappears just prior to birth, the LPA_1_ receptor shows rich expression in the postnatal brain, coincident with myelination [17]. The importance of LPA signaling for CNS development has been further confirmed in mice lacking the LPA_1_ receptor (LPA_1_-null mice), as these animals exhibited gross craniofacial alterations [18], smaller brain sizes and impaired cortical organization, among other abnormalities [11].

In the adult brain, LPA receptor expression is diminished, and the main receptor present is LPA_1_, and to a lesser extent, LPA_3_ and LPA_4_ are also observed; there are scarce data on the expression of LPA_5_ and LPA_6_ [2]. LPA receptors in the adult brain are present in glial cells and neurons [2], [15], [17], [19]–[20], suggesting a role for LPA in myelination and neuroinflammation [21], adult hippocampal neurogenesis [22] and memory [16], [23]. Nevertheless, the role for LPA in emotional behavior remains poorly defined. Most of what we currently know is derived from the analysis of transgenic mice lacking the LPA receptors, and LPA_1_-null mice have been the most studied transgenic mice thus far. LPA_1_-null mice are sensitive to both chronic and acute stress and exhibit increased anxiety-like responses and hippocampal-dependent memory deficits [24]–[30]. The phenotype of LPA_5_-null mice has been recently described and, on the contrary, involves the potentiation of spatial memory with anxiolytic-like responses across several tests [31]. These studies strongly suggest that LPA, acting through LPA receptors, regulates emotion. However, these studies have significant limitations, as the analyses were restricted to the function of a single LPA receptor type and, importantly, the potential effects of LPA signaling in adulthood are likely confounded through neurodevelopmental alterations present in LPA receptors-null mice [11].

Pharmacological studies are therefore required to understand the role of LPA as a modulator of emotional processes. To address this issue, we examined the behavioral profile of adult rats receiving intracerebroventricular (i.c.v.) infusions of LPA 18∶1. LPA 18∶1 is an abundant LPA species with high biological activity due to its strong affinity for the LPA receptors; thus, LPA 18∶1 is commonly used in most laboratories as a reagent for LPA receptor activation [7], [10], [32]. Moreover, we mapped the potential brain sites involved in the actions of LPA through an analysis of LPA 18∶1-induced c-Fos expression. The results clearly indicate that LPA modulates emotional behavior, a relevant finding for further analysis in the context of affective disorders in humans.

## Materials and Methods

### Ethics Statement

All the procedures involving the care and use of animals were conducted in adherence with the European Directive 2010/63/EU on the protection of animals used for scientific purposes and with Spanish regulations (Real Decreto 53/2013 and 178/2004, Ley 32/2007 and 9/2003, and Decreto 320/2010) for the use of laboratory animals and transgenic animals. Surgery was performed under equithesin anesthesia and all efforts were made to minimize animal suffering. The experimental research protocols included in the present study were evaluated and approved by the “Comisión de Ética e Investigación Sanitaria” of the Carlos Haya Hospital, Avenida Carlos Haya 82, 29010 Málaga, SPAIN, in his ordinary session of July 11th 2011. Ethical Commission resolution and additional information related to this Project can be obtained by addressing a letter to the President of the Ethical Commission.

### Animals

All experiments were performed on 9-week-old male Wistar rats (Charles Rivers Laboratories España, S.A., Barcelona, Spain) weighting 250–300 g. In addition to the rats, for the immunohistochemical analysis of LPA_1_ receptor distribution we used both male normal (wild-type) mice and male mice lacking the LPA_1_ receptor (LPA_1_-null mice), that served as a negative control for LPA_1_ receptor localization. The male homozygous maLPA_1_-null mice were derived from a colony (the *Malaga* variant of LPA_1_-null) developed in our laboratory and extensively described in our previous works [11], [22]. The animals were housed in a humidity (55%)- and temperature (22°C)-controlled vivarium on a 12 h light/dark cycle (lights on 8∶0 PM). Water and standard chow pellets (Prolab RMH 2500 from LabDiet®, St. Louis, MO, USA) were available *ad libitum* unless otherwise indicated.

### Drugs

1-Oleoyl-2-hydroxy-sn-glycero-3-phosphate sodium salt (LPA 18∶1) was obtained from Avanti Polar Lipids (Alabaster, AL, USA), dissolved in stock solutions at 100% ethyl alcohol, and diluted to the appropriate concentration for i.c.v. injection in sterile saline containing 0.15% ethyl alcohol at concentrations of 0 (i.e., vehicle solution), 0.4 or 2 µg/5 µL. To our knowledge, a few studies have administered LPA directly in the brain [16], [33]. However, those experiments did not provide clear information about the LPA dose required to exert significant effects, as highly heterogeneous LPA doses were employed (10 nmol, equivalent to 4.37 µg [33]; or 40 ng [16]), and the central administration method (i.c.v. or intra-hippocampal), the animal species (Balb/c mice or Long-Evans rats) or the variable analyzed (orofacial pain or long-term memory consolidation, respectively) varied. Therefore, doses of 0.4 and 2 µg of LPA were used in this study with the intention of representing a low-medium dose range. Because the preliminary results showed that 2 µg of LPA was sufficient to affect behavior and that 0.4 µg of LPA did not yield any effects, these doses were considered appropriate for our experiments.

### Surgery and i.c.v. Injection Procedure

Given the abundant distribution of the LPA receptors throughout the organism [2], the i.c.v. administration protocol was used to study the specific role of LPA in the brain, thereby avoiding confounding results derived from potential peripheral effects.

For i.c.v. injections and administration, we used a previously described protocol [34]–[35]. Stainless steel guide cannulae aimed at the left or right lateral ventricle were implanted in the rats. The animals were anesthetized with equithesin and placed in a stereotaxic apparatus (David Kopf Instruments Tujunga, CA, USA) with the incisor bar set at 5 mm above the interaural line. A guide cannula (7 mm and 23-gauge) was secured to the skull using two stainless steel screws and dental cement, and the opening was closed using 30-gauge obturators [36]. The implantation coordinates were 0.6 mm posterior to bregma, ±2.0 mm lateral, and 3.2 mm below the surface of the skull, according to the rat brain stereotaxic coordinates of Paxinos and Watson [37]. These coordinates placed the cannula 1 mm above the ventricle. After a 7-day postsurgical recovery period, the cannula patency was confirmed through the gravity flow of isotonic saline through an 8-mm long and 30-gauge injector inserted within the guide to 1 mm beyond the tip. This procedure was used to familiarize the animals with the injection technique (sham injection). The obturator was removed from the guide cannula, and an 8 mm injector (30 gauge stainless steel tubing), connected to 70 cm of calibrated polyethylene-10 tubing, was lowered into the ventricle. The tubing was subsequently raised until the flow was initiated, and 5 µL of drug or vehicle solution was infused over a 30–60 s period. The injector remained in the guide cannula for an additional 30 s to facilitate the diffusion of the solution and subsequently was removed. The stylet was immediately replaced. The guide cannulae, tubing and screws were purchased from Small Parts (Miami Lakes, FL, USA).

The i.c.v. cannula placements were evaluated after each experiment using dye injection. Only rats with proper i.c.v. placements were included in the data analysis.

### Behavioral Assessment

Behavioral testing was performed at 5 min after the injection of vehicle or LPA 18∶1 at 0.4 or 2 **µ**g doses subsequent to sessions with sham injections to familiarize the animals with the i.c.v. infusion procedure. Considering that information concerning the time required for the LPA to act in the brain is lacking, we selected a time interval of 5 min from administration to behavior, as this time has been demonstrated to be suitable after the i.c.v. administration of several modulators of anxiety and depression [38]–[40].

Each behavioral assessment was performed on different groups of rats; thus, the animals were not evaluated more than once. The groups comprised 7 to 12 animals depending on the test (the exact number of animals in each group is detailed below). Trained observers, unaware of the experimental conditions, scored the behavioral measures.

#### Elevated plus-maze and open-field test

**Figure 1:**
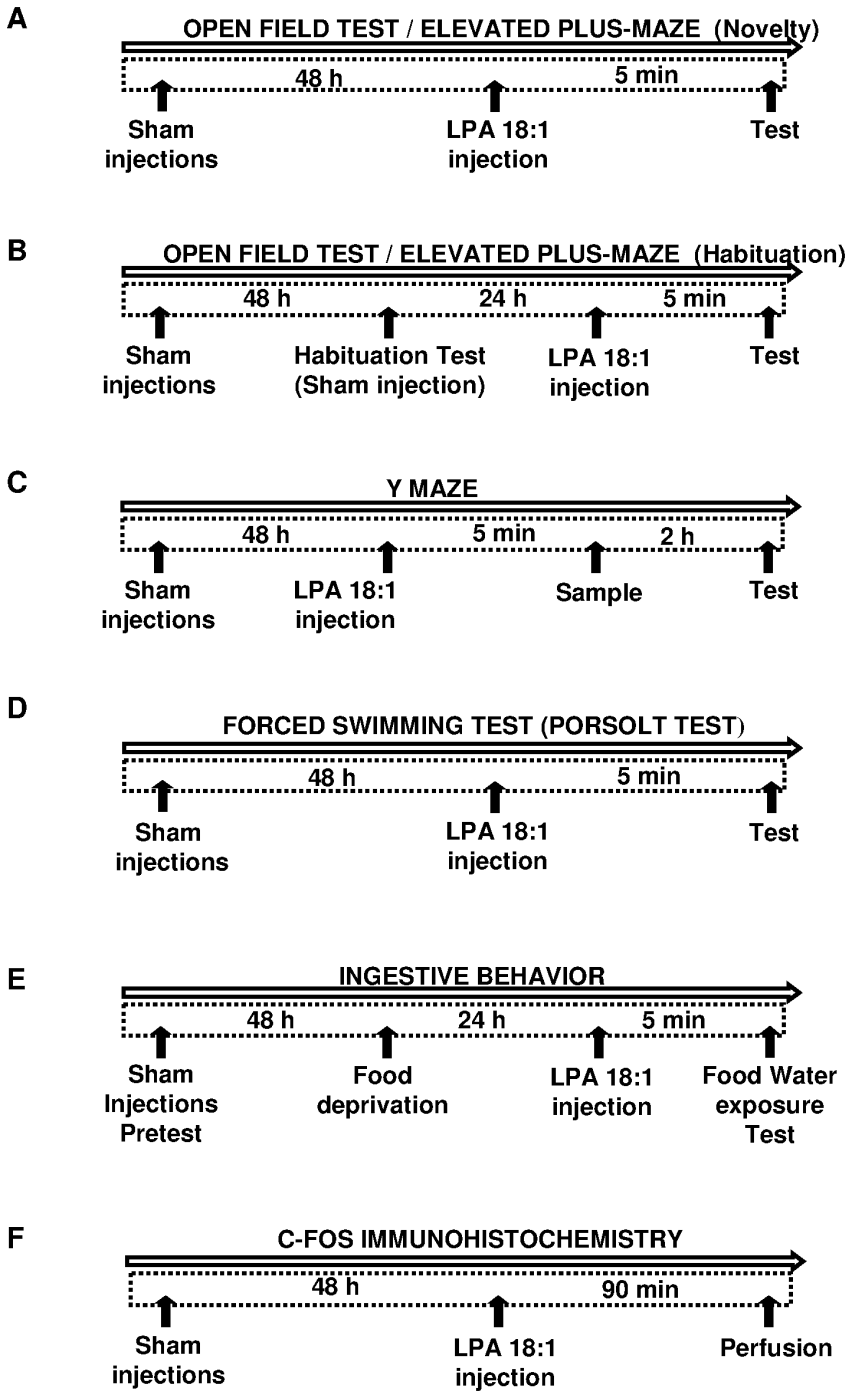
Time schedules and experimental designs. Male Wistar rats were sham injected at least two times prior to LPA treatment to familiarize animals with the i.c.v. procedure. The open field test (OFT) and elevated plus maze (EPM) were performed under novelty (A) and habituation (B) conditions after 5 min of LPA 18∶1 infusion. For habituation, the rats were sham injected and tested in both paradigms 24 h before LPA infusion. Rats were exposed to the sample trial of the Y maze (YMT) 5 min after LPA 18∶1 administration, and performed the test trial 2 h later (C). The forced swimming test (FST) was performed after 5 min of LPA 18∶1 infusion (D). Food and water intake were evaluated at different times in 24 h food-deprived animals after 5 min of LPA 18∶1 infusion (E). c-Fos immunoreactivity (IR) was performed through perfusion after 90 min of LPA 18∶1 infusion (F).

Exploratory and anxiety-like behaviors in a novel environment were assessed during one session in the elevated plus maze (EPM) or in the open-field test (OFT) (novelty condition, **Figure 1A**). Because familiarity with an environment reduces the threatening impact and profoundly changes the animals’ behavior [41]–[42], the effect of LPA 18∶1 administration was also tested on habituated animals previously exposed to both tests (habituation condition, **Figure 1B**).

The performance in the EPM was evaluated using a previously described method [43]. The apparatus comprised two wooden open arms and two enclosed arms (40 cm high walls) of the same size (50×10 cm), arranged with the arms of the same type opposite each other. The maze was elevated 50 cm above the floor, and the room was illuminated to 350 lux. For the novelty condition, 5 min after vehicle or drug injection, each animal was placed in the center of the maze, facing a closed arm, for a 5-minute session. For the habituated condition, the treated rats were previously exposed for 5 min to the EPM on the previous day. The total time spent in each arm and the number of total arm entries were scored. A total of 12 animals per experimental treatment (vehicle, 0.4 µg of LPA and 2 µg of LPA) were tested either under novelty or habituation conditions in the EPM.

Motor behaviors in the OFT were studied in an opaque open field (100×100×40 cm) using a floor marked with lines forming 20×20 cm squares, as previously described [43]–[44]. The field was illuminated using a ceiling halogen lamp regulated to 350 lux at the center of the field. On the experimental day, the animals were treated and placed in the center of the field for 15 min at 5 min after vehicle or drug injection. For the condition of habituation, the rats were placed in the field for 15 min on the day prior to testing. Locomotor activity (the number of lines that the animal crossed with all four paws) and the time spent in the center of the field (defined as the 9 quadrants in the middle of the apparatus, while the surrounding 16 quadrants were defined as the periphery) were assessed. A total of 12 animals per treatment were tested under the novelty condition in the OFT, while the habituation condition comprised 12 vehicle-treated animals, 11 animals in the 0.4 µg LPA group and 10 animals in the 2 µg LPA group.

#### Y maze test

The Y maze test (YMT) was used to assess preference for novelty (**Figure 1C**). The sample trial took place 5 min after vehicle or drug (0.4 µg or 2 µg LPA) administration. Rats were placed in the center of the apparatus with one of the arms closed, and allowed to explore the remaining two arms for 2 min. Rats were then returned to their homecage for 2 hours. In the test trial, rats were again placed in the center of the Y maze and allowed to explore freely for 2 min, but all the three arms were opened. The ‘novel arm’ was defined as the one that was closed during the sample test (and thus previously unexplored), and was scented with vanilla to enhance its novelty. The arm assigned as ‘novel’ was changed across rats to control for any intrinsic preference. The first arm the rat chose to entry (novel vs not novel), the total time spent in the novel arm and the total number of arm entries were scored during the test trial. The number of animals used for this test were 11 rats for the vehicle group and 12 for each LPA-treated conditions.

#### Forced swimming test

Behavioral despair in stressful and inescapable situations was assessed in the forced swimming test (FST) [45]–[46] (**Figure 1D**). In this case, 5 min after drug administration i.c.v., the rats were placed for 6 min in a glass cylinder (40 cm diameter and 80 cm high) filled with water (23±1°C) to a height of 60 cm. Immobility, defined as the absence of directed movements of the animal’s head and body, was estimated during the last 4 min of the test. The animals used for the FST included 7 rats treated with vehicle, 7 rats administered 0.4 µg LPA and 8 rats administered 2 µg LPA.

#### Food intake experiment

The food and water intake were evaluated to study the effect of LPA 18∶1 on ingestive behavior (**Figure 1E**). Feeding behavior was analyzed in 24 h food-deprived animals with free access to water. These rats were first habituated to handling, and 48 h before testing, the bedding material was removed from the cage, and a small can containing food pellets was placed inside the cage for 4 h [36], [47]. In the food intake test, LPA 18∶1 was administered i.c.v., and the rats were returned to their individual home cages at 5 min before being given access to food and water. Subsequently, a measured amount of food (25 g) and water (250 mL) was placed in the cages (t = 0). The food pellets and food spillage were weighed and recorded at 30, 60, 120, and 240 min, and the amount of water consumed was also measured at 240 min. The cumulative food intake (g) was calculated using these data. A total of 12 rats treated with vehicle, 10 rats treated with 0.4 µg LPA and 11 rats treated with 2 µg LPA were used for this experiment.

### c-Fos Immunohistochemistry and Quantification

Because the 2- µg dose of LPA 18∶1 was most effective to modulate behavior, we aimed to assess the effects of LPA on neuronal activation through the expression of the early immediate gene *c-fos* (Figure 1F). A total of 4 rats per treatment were administered saline, vehicle or 2 µg of LPA 18∶1 and then perfused at 90 min after the i.c.v. injection. The animals were deeply anaesthetized with equithesin (3 mL/kg) and perfused via the ascending aorta with cold physiological saline solution followed by a cold formalin buffer (4% paraformaldehyde in 0.1 M phosphate buffer, pH 7.4). The perfusion was continued for 5 min, and the brains were postfixed in the same fixative for 2 h. The brains were subsequently transferred to phosphate-buffered saline (PBS, pH 7.4) containing 30% sucrose for cryoprotection. Coronal sections (40 µm) of the brain were cut at −20°C using a cryostat. Free-floating sections were processed for immunohistochemistry using an anti-c-Fos rabbit polyclonal antibody (Santa Cruz Biotechnology Inc., Santa Cruz, CA, USA), according to a previously described protocol [48].

For quantification of c-Fos immunoreactivity (IR), the unbiased stereological estimation of the density of c-Fos IR nuclei in the dorsal periaqueductal gray matter (DPAG) was obtained using the optical dissector principle and random systematic sampling [49]. The number of c-Fos IR nuclei was estimated using 100 optical dissectors in each case. Each optical dissector was a 43.4×43.4 µm (1,883.56 µm^2^) sampling frame with exclusion lines. The starting point for counting was set at 5 µm below the surface, and the labeled nuclei were focused as the optical plane moved 15 µm through the tissue. The quantification was performed using a 100x oil-immersion lens on an Olympus BX 51 microscope, equipped with a JVC (TK-C1480E) camera and a computer-assisted stereological toolbox (CAST-Grid software package from Olympus Europa Holding GmbH, Hamburg, Germany). The number of cells per unit of volume was calculated for each animal as the number of c-Fos IR nuclei per mm^3^ using the following formula: Nv = ∑Q^−^_d/_∑V_dis_, where Nv = numerical density; ∑Q^−^_d_ = total number of c-Fos IR nuclei counted; and ∑V_dis_ = total number of dissectors applied multiplied by V_dis_. V_dis_ = S_d_ × H_d_, where S_d_ = area of the dissector grid (counting frame) and H_d_ = depth of the dissector.

### LPA_1_ Receptor Immunohistochemistry

The rats were perfused as described above. For the maLPA_1_-null mice, a periodate-lysine-paraformaldehyde solution was used. The brains were processed for immunohistochemistry using an anti-LPA_1_ rabbit polyclonal antibody (Pierce-Thermo Scientific Inc.) according to a previously described protocol [11]. Coronal sections, including the PAG, were used for confirmation of the presence of the LPA_1_ receptor.

### Statistical Analyses

All data for graphs and tables are expressed as the mean ± standard error of the mean (SEM). Statistical analyses were performed using GraphPad Prism version 5.04 (GraphPad Software Inc., San Diego, CA, USA). For the behavioral experiments, the significance of the differences between groups was evaluated using one- (EPM, OFT, YMT and FST) and two- (food intake) way analysis of variance (ANOVA) followed by a *post hoc* test for multiple comparisons (Bonferroni). The percent of rats that first entered the novel arm in the YMT was analyzed by a Chi-square test. For quantification of c-Fos expression, the DPAG data were statistically analyzed using the Kruskal-Wallis one-way ANOVA by ranks followed by Dunn’s *post hoc* test for multiple comparisons. A (p)-value below 0.05 was considered statistically significant.

## Results

### LPA 18∶1 Induces Anxiety-like Responses in the EPM Under Novelty Conditions

**Figure 2:**
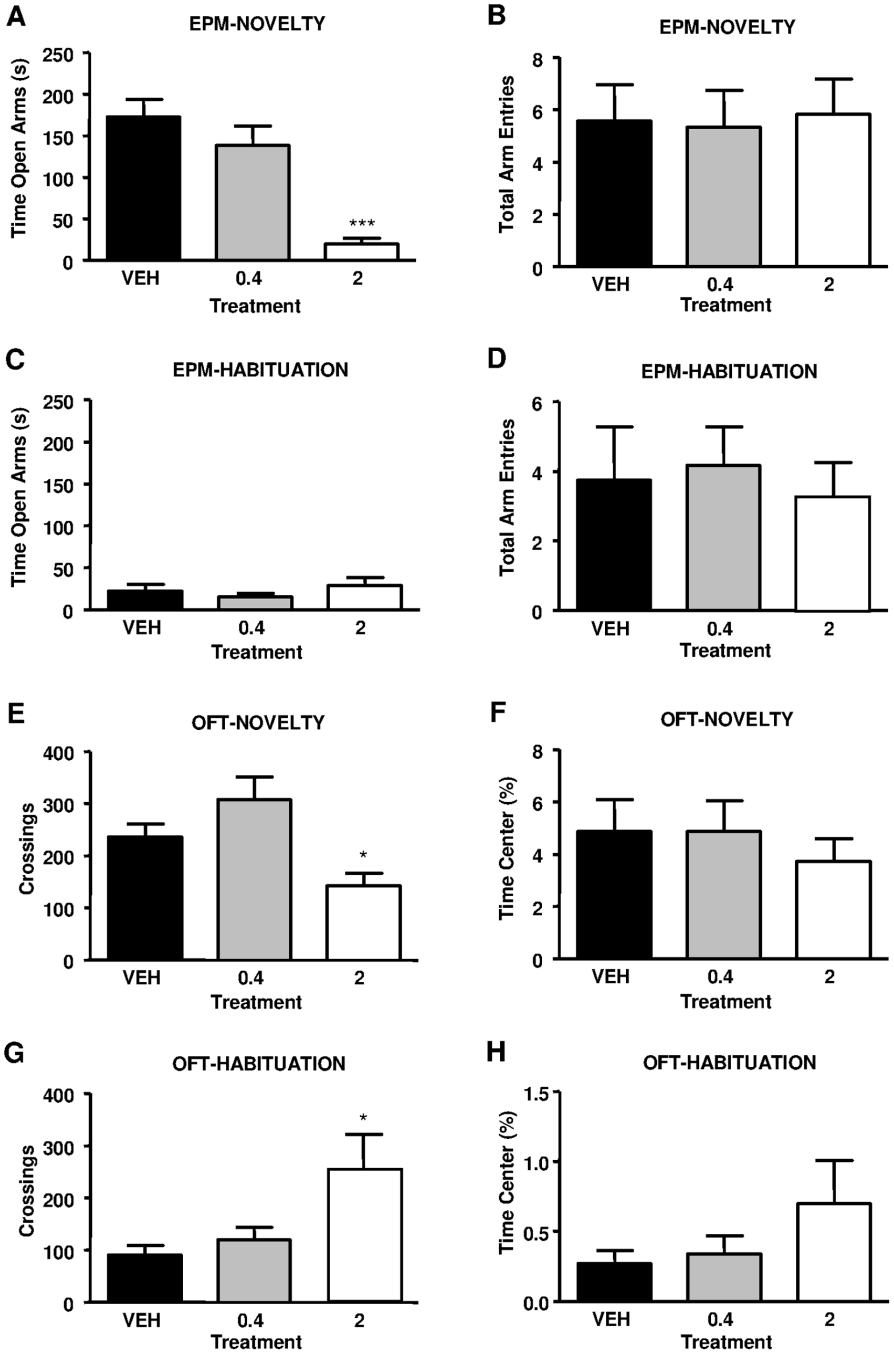
Wistar rats were studied in the elevated plus maze (EPM) and open field test (OFT) under novelty and habituation conditions following LPA 18∶1 infusion at doses of 0, 0.4 and 2 µg. In the EPM, the time (s) exploring the exposed arms (A) and the total number of arm entries (B) were evaluated under novelty conditions. Similarly to the novelty conditions, the time exploring the open arms (C) and the total arm entries (D) were evaluated in animals previously habituated to the EPM (A). Locomotor activity was measured based on the number of crossings (E) and the time (%) spent in the center of the field (F) under novelty conditions. Again, both the number of crossings (G) and the percentage of time spent in the center (H) were evaluated under habituation conditions. The bars are the means ± SEM (n = 7–12 animals per group). The data were analyzed using one-way ANOVA. *p<0.05 and ***p<0.001 denote significant differences versus the vehicle-treated group, determined using Bonferroni’s *post-hoc* test.

To explore the emotional alterations induced through LPA 18∶1, we analyzed behavioral performance in the EPM, a standard test for anxiety-like behaviors. When LPA 18∶1 was centrally injected under novelty conditions, such infusion produced decreased exploration time in the open arms of the EPM (F_2, 33_ = 18.520, P<0.0001) (**Figure 2A**). The time in the open arms was reduced approximately 89% in rats administered 2 µg of LPA 18∶1 compared with vehicle-treated rats (***p<0.001), but no significant reduction was observed in the 0.4 µg group. However, differences in the total arm entries were not detected among the treatments (F_2, 33_ = 0.033, P = 0.9679) (**Figure 2B**). Therefore, locomotor deficits likely do not reflect the differences in the open arms time. Complementary to the novelty conditions, LPA was also injected in rats previously exposed to the EPM (habituated to the maze), and we did not detect any changes in the time of open arm exploration for any of the LPA-administered rats (F_2, 33_ = 0.867, P = 0.4289). As shown in **Figure 2C**, habituation to the EPM produced a drastic decrease in the time of open arm exploration for vehicle- and 0.4 µg LPA-treated animals (p<0.001) compared with novelty conditions. Therefore, all habituated groups reached levels similar to those of rats treated with 2 µg of LPA, which showed no differences between novelty and habituated conditions. Regarding the total arm entries with habituated rats, we also detected no differences among treatments (F_2, 33_ = 0.138, P = 0.8715) (**Figure 2D**). Thus, LPA 18∶1 reduced open arm exploration under novelty conditions and had no effect under habituated conditions in the EPM.

### LPA 18∶1 Reduces Locomotor Activity in the OFT Under Novelty Conditions

During the behavioral characterization of animals receiving LPA 18∶1, we measured spontaneous locomotor activity using the OFT. Under novelty conditions, one-way ANOVA indicated that infusions of LPA 18∶1 produced a significant effect on the number of crossings in the OFT (F_2, 33_ = 6.551, P = 0.0026) (**Figure 2E**). Indeed, the infusion of 2 µg of LPA 18∶1 resulted in a significant decrease of approximately 40% in locomotion in the OFT compared with vehicle-treated rats (*p<0.05). Rats treated with 0.4 µg of LPA showed locomotion similar to the vehicle group. In addition to the number of crossings, we measured the time spent in the center area of the OFT (**Figure 2F**). We observed no differences in this variable of the OFT among treatments (F_2, 33_ = 0.350, P = 0.7061), as animals from all groups barely entered the center. When LPA 18∶1 was injected in animals experienced with the OFT (habituated to the test), we observed significant effects on the number of crossings by treatment (F_2, 32_ = 4.177, P = 0.0251) (**Figure 2G**). In contrast to novelty conditions, the administration of 2 µg of LPA 18∶1 produced a significant increase of 180% in locomotion compared with the vehicle treatment (*p<0.05) under familiarity conditions with the OFT, while locomotion in the 0.4 µg LPA group remained similar to that of the vehicle-treated group. As shown in **Figure 2H**, we did not detect any changes in the time spent in the center of the OFT under habituation conditions (F_2, 32_ = 1.449, P = 0.2508). Therefore, while locomotor activity was reduced through LPA 18∶1 under novelty conditions, locomotion was increased in rats habituated to the OFT.

### LPA 18∶1 Impairs Preference for Novelty in the YMT

**Figure 3:**
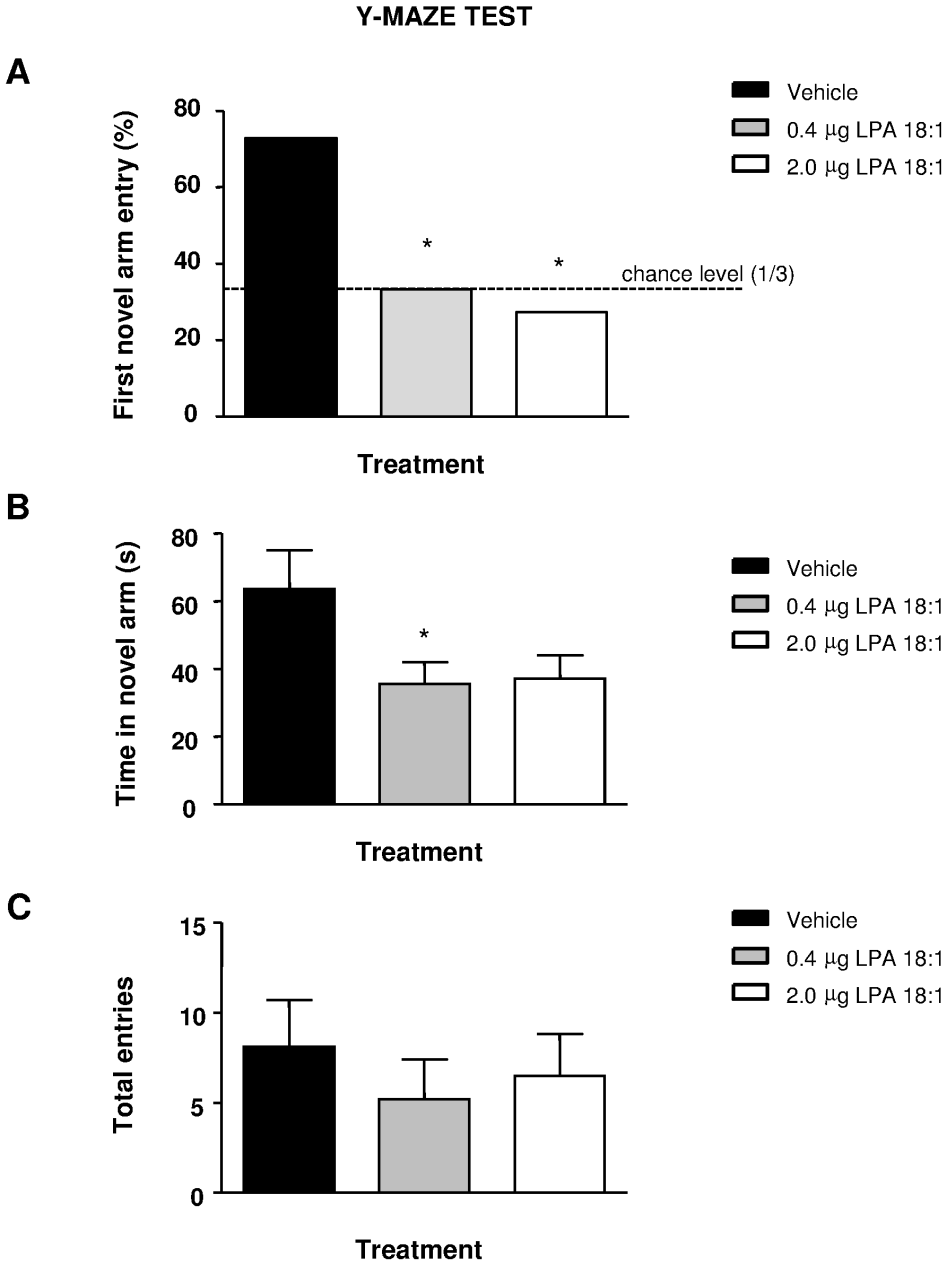
Rats were studied for novelty recognition in the Y maze (YMZ). LPA 18∶1 infusion at doses of 0, 0.4 and 2 **µ**g was carried out 5 min before the sample trial, and the test trial was performed 2 h later. The percent of rats of each group that first entered the novel arm (A), the total time of novel arm exploration (B) and the total arm entries (C) in the test trial were avaluated. The bars are the means ± SEM (n = 11–12 animals per group). The data were analyzed using a Chi-square test (A) or an one-way ANOVA followed by Bonferroni’s *post-hoc* tests (B–C). *p<0.05 denote significant differences versus the vehicle-treated group. The comparison of the 2 **µ**g and the vehicle group in (B) was significant at p = 0.0558.

Results of the test trial revealed that rats treated with 0.4 or 2 µg of LPA 18∶1 did not first entry the novel arm as much as the vehicle-treated rats (Chi-square test: P = 0.0330 for the 2.0 dose and P = 0.0487 for the 0.4 dose, vs vehicle group), showing a chance performance (**Figure 3A**). Moreover, while the vehicle group spent more than half of the trial duration within the novel arm, the total time of novel arm exploration was reduced by the LPA treatment (F_ 2, 34_ = 3.445, P = 0.0442), which was significant for the 0.4 µg dose (*p<0.05) and an almost significant for the 2 µg group (p = 0.0558) (**Figure 3B**). No differences were found in locomotion assessed as the total arm entries (F_2, 34_ = 0.372, P = 0.6926) (**Figure 3C**).

### LPA 18∶1 Increases Immobility in the FST

**Figure 4:**
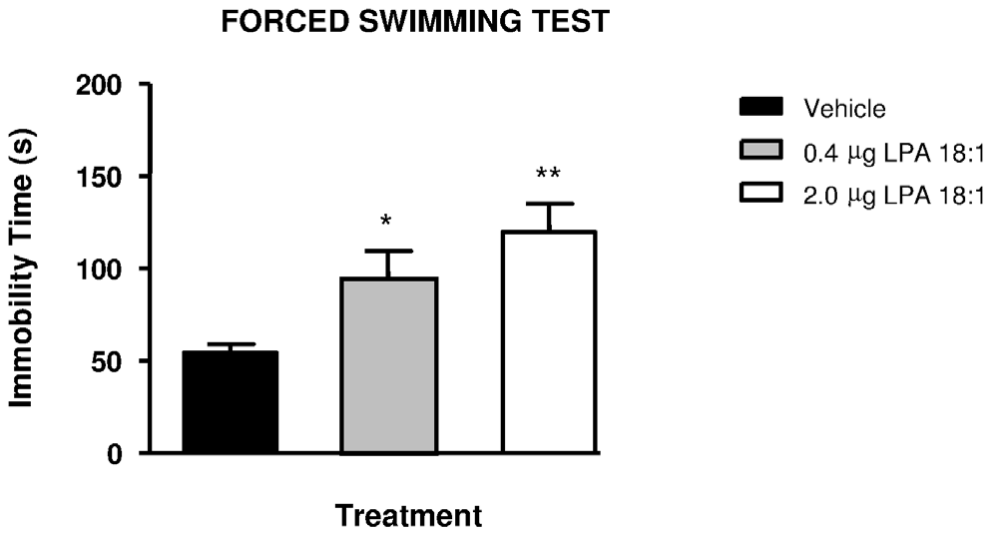
Wistar rats were studied in the forced swimming test (FST) following LPA 18∶1 infusion at doses of 0, 0.4 and 2 µg. The immobility time (s) was evaluated in these animals. The bars are the means ± SEM (n = 8 animals per group). The data were analyzed using one-way ANOVA. *p<0.05 and **p<0.01 denote significant differences versus the vehicle-treated group, determined using Bonferroni’s *post-hoc* test.

Immobility or floating behavior in the FST has been associated with depressive-like behaviors. As observed in **Figure 4** **,** one-way ANOVA revealed that immobility time was significantly affected after treatment in the FST (F_2, 19_ = 6.428, P = 0.0074). LPA 18∶1 infusions increased immobility in the FST in a dose-dependent manner, resulting in a significant increase of 75% in rats treated with 0.4 µg (*p<0.05) and of 120% in rats treated with 2 µg (**p<0.01) of LPA 18∶1 compared with vehicle-treated rats.

### LPA 18∶1 does not Affect Feeding Behavior

The feeding behavior was evaluated in 24 h food-deprived rats treated with LPA 18∶1 infusion at 5 min prior to food exposure and food consumption, and this measurement was obtained at different times during 240 min (**Table 1**). The data were analyzed using two-way ANOVA (time and treatment were the factors), revealing a non-significant effect of treatment on cumulative food intake (F_2, 84_ = 0.8538; P = 0.4295), with no interaction with time (F_6, 84_ = 0.1053; P = 0.9956). As expected, the time factor reflected the increase in food intake (F_3, 84_ = 26.42; P<0.0001). In addition to the feeding behavior, water consumption was also evaluated at 240 min, and we observed no differences among groups after the corresponding one-way ANOVA (F_2, 21_ = 0.1867; P = 0.8311). Thus, 0.4 and 2.0 µg of LPA 18∶1 did not affect eating and drinking behavior in 24 h food-deprived animals.

**Table 1:** Time-course effects of i.c.v. administration of LPA 18∶1 or vehicle on food and water intake in 24 h food-deprived Wistar rats.

Treatment	Food Intake (g)				WaterIntake (mL)
	30 min	60 min	120 min	240 min	240 min
Vehicle	2.5±0.4	5.6±0.6	6.6±0.7	7.8±0.9	34.4±2.5
LPA 18∶1 (0.4 µg)	2.4±0.4	5.3±0.7	6.4±0.9	7.7±1.1	32.9±2.6
LPA 18∶1 (2 µg)	2.2±0.5	5.0±0.7	6.1±0.8	6.6±0.7	32.2±2.7

^1^ Data are means ± SEM of 8 determinations per group.

### LPA 18∶1 Enhanced c-Fos Expression on the DPAG

**Figure 5:**
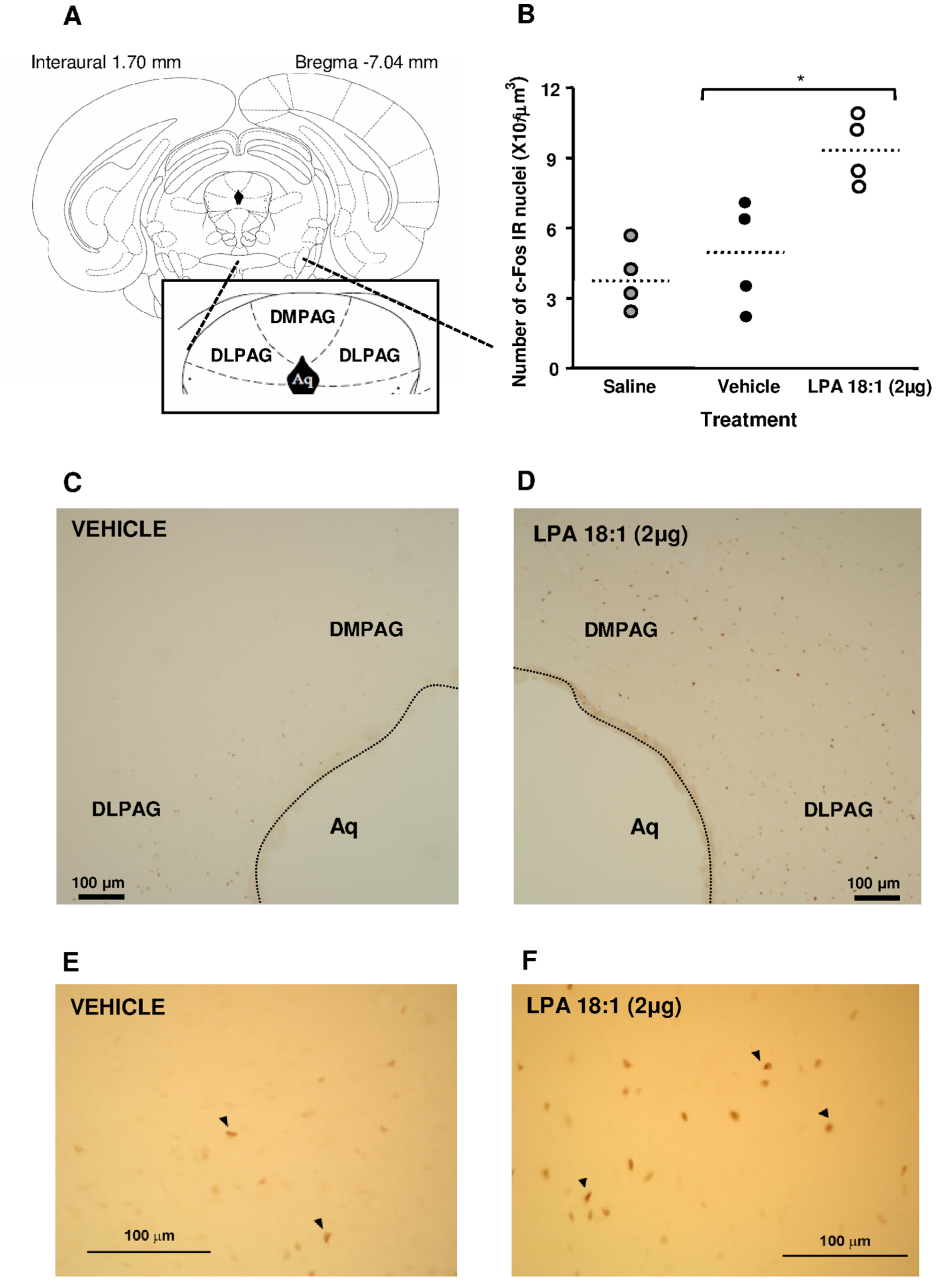
c-Fos immunohistochemistry in the rat dorsal periaqueductal gray matter (PAG) following LPA 18∶1 infusion at doses of 0 and 2 µg. Analyses were performed in the dorsomedial and dorsolateral divisions of the PAG (**A**). Stereological quantification of c-Fos immunoreactive (IR) nuclei in the DPAG (**B**). Low magnification microphotographs for c-Fos immunohistochemistry are depicted for the vehicle (**C**)- and LPA 18∶1 (**D**)-treated groups. In addition, high magnification images for the vehicle (**E**)- and LPA 18∶1 (**F**)-treated groups are shown. Each point represents the total number of immunopositive nuclei per animal. The dotted lines are medians (n = 4 animals pr group). The data were analyzed using Kruskal-Wallis one-way ANOVA. *p<0.05 denotes significant differences versus the vehicle-treated group, determined using Dunn’s *post-hoc* test. The arrowheads indicate immunopositive nuclei for c-Fos.

An initial preliminary stereological analysis was performed in the prefrontal cortex, amygdalar complex (central, basolateral and anterior division), entorhinal cortex, hippocampal formation (dentate gyrus, CA1 and subiculum subdivisions), DPAG and hypothalamic paraventricular nuclei [37]. This analysis indicated that only the DPAG (**Figure 5A**) exhibited marked differences in c-Fos expression (**data not shown**). Thus, a complete stereological analysis was performed in this brain area. Central injections of 2 µg of LPA 18∶1 affected c-Fos IR in the DPAG compared with saline and vehicle treatment (H = 7.538; P = 0.0231). The *post hoc* test indicated that c-Fos IR in LPA 18∶1-treated rats significantly increased compared with c-Fos IR in saline- or vehicle-treated rats (*p<0.05) (**Figure 5B**).

As shown in **Figure 5C–F**, c-Fos IR increased 94% in the dorsomedial and dorsolateral areas of the periaqueductal gray matter (PAG) when the LPA 18∶1 treatment was compared with the vehicle treatment.

### Location of LPA_1_ Receptors on the PAG

**Figure 6:**
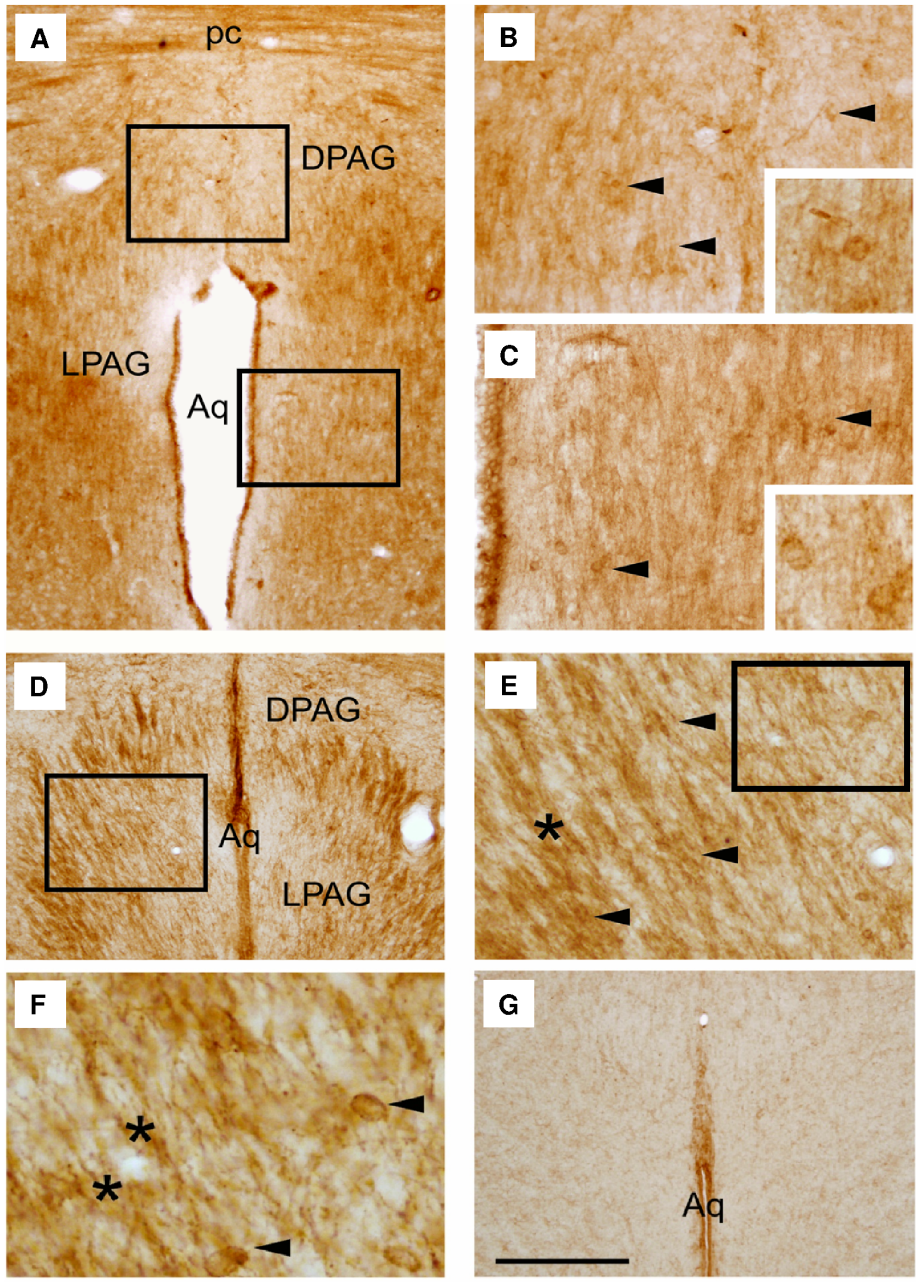
Distribution of LPA_1_ receptor immunoreactivity (IR) in the periaqueductal gray matter (PAG) of adult rats and mice. Coronal brain section showed the LPA_1_ receptor IR in the PAG of the adult Wistar rat (**A**). Representative micrographs from image **A** show that LPA_1_ IR is distributed in the dorsal (DPAG) (**B**) and lateral (LPAG) (**C**) area of rats. Comparable coronal sections of the PAG area in wild-type mice showed LPA_1_ IR in the DPAG and LPAG (**D**). Enlarged from image **D**, neuronal cell bodies and fibers display the expression of LPA_1_ (**E**). Magnified from image **E**, cell bodies and fibers are observed in detail (**F**). In maLPA_1_-null adult mice, LPA_1_ receptor IR is absent, and only the aqueductal ependyma exhibits a non-specific reaction (**G**). The arrowheads and asterisks indicate cell bodies and fibers, respectively. Scale bars: **A**, **D**, **G**, 500 µm; **B**, **C**, **E**, 180 µm; **F**, 50 µm.

Coronal brain sections showing the general distribution of LPA_1_ receptor IR in the PAG of adult rats, wild-type and LPA_1_-null mice are shown in **Figure 6**. In rats, most of the stain intensity was distributed in the DPAG and lateral PAG (LPAG) area, showing LPA 18∶1-induced c-Fos IR expression (**Figure 6A–C**). The pattern of LPA_1_ receptor expression in both rats and wild-type mice was similar, as observed in **Figure 6D–F**. However, maLPA_1_-null mice exhibited only nonspecific IR expression in the ventricular wall (**Figure 6G**). Thus, LPA_1_ expression was observed in neuronal cell bodies (arrowheads) and fibers (asterisks) in both rodent models.

## Discussion

In this study, we have addressed the effects of the central administration of LPA 18∶1 on emotional-like behaviors. The results were consistent with an anxiogenic- and depressive-like phenotype induced through LPA, yielding increased c-Fos activity in the DPAG.

In this regard, animals injected with 2.0 µg of LPA 18∶1 demonstrated reduced time in the open arms in the EPM and decreased exploratory activity in the OFT, responses that are both indicative of a high emotional arousal. The LPA-treated animals, however, did not differ from controls with regard to the time spent in the OFT center, another measure of anxiety that is expected to decrease under anxiogenic-like conditions. This divergent result likely reflects a ‘floor effect’. While the vehicle-treated animals visited the open arms in the EPM, these animals barely entered the center of the OFT (spending less than 5% of the total test time there), and thus, this measure could hardly be reduced to show any anxiogenic-like effect, suggesting that the OFT could have elicited greater aversion in the rats than the EPM testing. However, those tests may not be directly comparable as they might reflect different aspects of emotion, considering that pharmacological studies often indicate a scarce correlation among the anxiety-like behaviors measured in different paradigms (see [50]–[51] for a review of this issue). Another interesting finding is that the anxiogenic-like phenotype was limited to novelty conditions, as no anxiety-like behaviors were induced through LPA when administered to animals previously habituated to the tests. Exposure to a novel context generates strong behavioral and stress responses that are notably reduced when the environment becomes familiar and the threatening impact is attenuated [41]–[42]. Therefore, the anxiogenic-like effect of LPA was restricted to the more stressful, unfamiliar environment, a similar profile to that described for anxiety-inducing peptides, such as the corticotropin-releasing factor (CRF) [52]. Moreover, the altered behavior of the LPA-treated rats tested on the YMT, where they did not prefered a novel arm scented with vanilla, suggests a defective novelty recognition or motivation for novelty, an aspect that may be sensitive to emotion.

The administration of LPA 18∶1 also induced a dose-dependent increase of immobility in the FST. This test estimates behavioral despair in stressful and inescapable situations, reflecting depression-like behavior [45]–[46]. This result is not surprising, as a high comorbidity between anxiety and depression has been observed, and manipulations that alter anxiety have frequently paralleled the effects on depressive-like behavior [54]–[57]. While both anxiety and depression-like behaviors were modulated by LPA signaling, the effect of LPA was not associated to alterations in feeding under food deprivation conditions. Notably, the environment where the feeding test was performed was highly familiar to the rat (i.e., its home cage), differing from other common paradigms, such as the novelty-induced hypophagia, in which a reduction in feeding is observed due to the exposure to a novel and anxiogenic environment [53]. Therefore, the feeding test in our study investigated the motivation to eat in the absence of any aversive or conflictive situation, in contrast with the other behavioral tests employed in this study (i.e., the EPM, the OFT and the FST). Interestingly, this dissociation suggests a separate circuit for LPA action on emotion and motivated behavior, such as feeding, consistent with previous data showing the independence of the mechanisms supporting anxiety and food intake [54].

Consistent with the anxiogenic-like phenotype, the histological study showed that LPA 18∶1 induced neuronal activation in the DPAG, evidenced through increased c-Fos protein expression in this brain area. The dorsal gray matter, particularly the dorsal division, is a key station in the integration of emotional processing of aversive stimuli (i.e., fear and panic responses) and nociception [55]–[56], where sensory and stress information elicits fight or flight reactions. The dorsal gray matter also contains neurons that respond to major stress hormones [57], and direct pharmacological manipulations with drugs affecting serotonin transmission [55]–[56], [58]–[59], GABAergic receptors [60], CRF inputs [61] or BDNF signaling [62] that result in anxiolytic or panicolytic responses. It remains unknown whether the LPA modulates these transmitter systems, as the only available evidence relies on the neurochemical abnormalities described in LPA_1_-null mice [63]–[64]. In any case, the recent description of the modulatory role of LPA in excitatory neurotransmission [14] suggests that LPA actions in the adult brain might be extended to the regulation of synaptic dynamics, beyond the classical concept of the modulation of neurogenesis and glial function.

The signaling pathways through which the LPA modulates emotional behavior remain to be elucidated. A potential mechanism involves the direct effect of LPA on the LPA_1_ receptors in the DPAG, identified through immunohistochemistry in both fibers and neuron-shaped cells within this area. The LPA_1_ receptor is a strong candidate to mediate the central effects of LPA on emotion. Indeed, LPA_1_ is the most abundant LPA receptor in the adult brain, expressed in both glial cells and neurons [8], [15], [17], [19], and is one of the main targets of LPA 18∶1 [2], [5], [10]. Moreover, the LPA_1_ receptor activates several intracellular signaling pathways mediated through Rho, Phospholipase C, Ras and Phosphatidylinositol 3-kinase proteins [7], signaling cascades that could regulate the expression of early immediate genes, such as c-Fos [65]–[67], and that have implications for anxiety [68]–[69]. As we previously reported, the involvement of the LPA_1_ receptor pathway in emotion is also consistent with the emotional alterations present in LPA_1_-null mice, as revealed through reduced activity in the OFT and increased anxiety-like behavior in the EPM and hole-board tests [27], [29]. Moreover, LPA_1_-null mice are very sensitive to acute stress, displaying an abnormal elevation of both corticosteroid levels and c-Fos activity in the amygdala [30]. After chronic stress, LPA_1_-null mice show hypocortisolemia, and the effects of chronic stress on spatial memory, adult hippocampal neurogenesis and hippocampal oxidative stress are aggravated in these mutants [25], [28].

Nevertheless, while mice lacking the LPA_1_ receptor show an anxiogenic-like phenotype, here we report anxiogenic changes after LPA administration, which presumably stimulates the LPA_1_ receptor. This somewhat contradictory finding could reflect profound developmental alterations in LPA_1_-null mice, as the LPA_1_ receptor plays a critical role during brain development [11]. Thus, LPA_1_-null mice show anatomical alterations in the limbic system, as revealed through reduced neuronal density and a lack of calcium-binding proteins in the basolateral amygdala, a key structure for emotional processing [30]. Moreover, the life-long absence of the LPA_1_ receptor likely leads to unknown neuroadaptive responses, such as an abnormal regulation of other receptors for LPA. These observations highlight the importance of performing pharmacological studies to understand the role of both the LPA and the LPA_1_ receptor on emotion and behavior, as the pharmacological modulation of these pathways in normal animals might yield different results than studies in mice with targeted mutations in LPA receptors, where neurodevelopmental alterations affect the outcome. However, while the participation of the LPA_1_ receptor seems highly likely, we cannot rule out the involvement of additional LPA receptors to explain the findings of this study. The LPA_2–4_ receptors show expression in the adult brain, although the presence of these molecules is low compared with the LPA_1_ receptor [2], and no data support the involvement of LPA_2–4_ receptors in behavior. The expression of the LPA_5_ receptor in the adult brain remains to be investigated, but intriguingly, the potential role for this receptor in emotion has been suggested by the anxiolytic-like phenotype of LPA_5_–null mice, which contrasts with that observed in LPA_1_-null mice [31]. The combined administration of LPA with selective inhibitors of LPA receptors is necessary to reveal the requirement of these molecules for LPA-induced effects.

No drugs targeting the LPA system are currently being considered as anxiolytic agents or antidepressants because few studies have shown the pharmacological modulation of the LPA system to investigate emotion or other behavior. Moreover, mice endophenotypes derived from genetic manipulations of the LPA signaling systems are just beginning to emerge, and no clinical counterpart studies in the context of the neuropsychopharmacological basis of psychiatric diseases have been published. However, the contribution of the LPA signaling system to neuropsychiatric disorders has been clearly shown, since previous studies have demonstrated a role for LPA in emotional memory. Dash and colleagues infused LPA bilaterally in the rat hippocampus after a training session in the Morris water maze and observed an increased memory consolidation that was dependent on the Rho pathway [16]. Recently, the LPA_1_ receptor has been associated with emotional learning, as the post-training i.c.v. administration of the LPA_1_ antagonist Ki16425 prevented the extinction of a fear-conditioned memory [30]. Therefore, the present study is the first to show the ability of LPA to modulate unconditioned anxiety-like responses and depression-like behaviors. These results support a role for drugs targeting the LPA system as a potential pharmacological approach to treat both anxiety and depression. Moreover, the LPA system might mediate the effect of currently known drugs to treat mood and anxiety disorders, which should be further investigated in future studies.
